# Intravesical Migration of Missed Intrauterine Device Associated with Stone Formation: A Case Report and Review of the Literature

**DOI:** 10.1155/2015/581697

**Published:** 2015-07-13

**Authors:** Mücahit Kart, Turgay Gülecen, Murat Üstüner, Seyfettin Çiftçi, Ufuk Yavuz, Cüneyd Özkürkçügil

**Affiliations:** ^1^Department of Urology, Hendek State Hospital, Sakarya, Turkey; ^2^Department of Urology, Sandıklı State Hospital, Afyon, Turkey; ^3^Department of Urology, Derince Training and Research Hospital, Kocaeli, Turkey; ^4^Department of Urology, Sivas State Hospital, Sivas, Turkey; ^5^Department of Urology, Karaman State Hospital, Karaman, Turkey; ^6^Department of Urology, Kocaeli University School of Medicine, Kocaeli, Turkey

## Abstract

Intrauterine device is the most widely used method of reversible contraception. It may cause various complications including perforation of uterus. In this case, 44-year-old woman was presented with lower urinary tract symptoms after six years of insertion. Patient has no remarkable physical or laboratory finding but abdominal ultrasound revealed a 27 mm hyperechogenicity, suggestive of foreign body or calculus on the posterior bladder wall which was removed endoscopically. This case highlights the need of immediate and periodic evaluation of women with intrauterine device to avoid missing serious complications.

## 1. Introduction

Currently, intrauterine device (IUD) is the most widely used method of reversible contraception because of its high efficiency and low complication rate, used on over 100 million women [1–3]. The use of IUD may cause complications from slight discomfort to sepsis leading to death [4]. Uterine perforation by an IUD is an uncommon complication; incidence is 1–3 in 1000 applications [5]. However, transvesical migration or misplacement of an IUD is a very rare complication with a high ratio of calculi formation [3, 6].

The aim of this case report is to show that persistent lower urinary tract symptoms (LUTS) of a woman with IUD may be associated with intravesical migration and stone formation in bladder.

## 2. Case Report

A 44-year-old woman was admitted to our outpatient clinic presenting with dysuria and intermittent hematuria for 2 years. She had a medical history of insertion of an IUD inserted 9 years ago. Three months after the insertion of IUD, she fell pregnant unexpectedly. It was her third pregnancy when she was 35 years old and previously she had two children who were 9 and 10 years old at that time. At the visit for pregnancy, the string of the device had not been detected by her gynecologist and it was assumed that IUD had been expelled spontaneously. She had continued her pregnancy and had a normal vaginal delivery without complication. Forty days after delivery, a second IUD had been inserted for contraception. After insertion of the second IUD she had no complaint in the following six years. The second IUD was removed 2 years before her presentation by her gynecologist because of the persistent urinary symptoms which was of newly onset. Physical examination at that time did not show any remarkable finding. Urinalysis was indicative of pyuria and hematuria and urine culture was negative. Abdominal ultrasonography revealed an echogenic intravesical lesion measuring about 27 mm with distal acoustic shadow suggestive of a foreign body or calculus. Plain radiography of the pelvis was not informative enough to show details of a foreign body.

**Figure 1:**
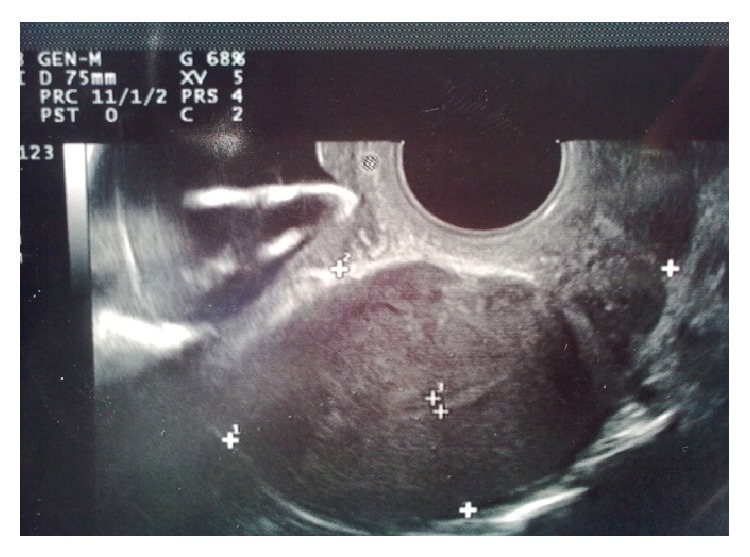
Transvaginal USG view of IUD.

**Figure 2:**
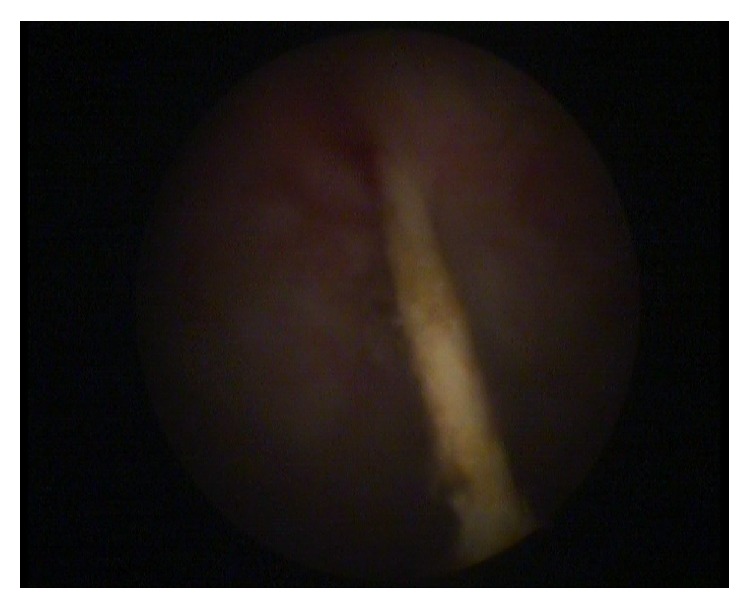
Cystoscopic view of IUD.

**Figure 3:**
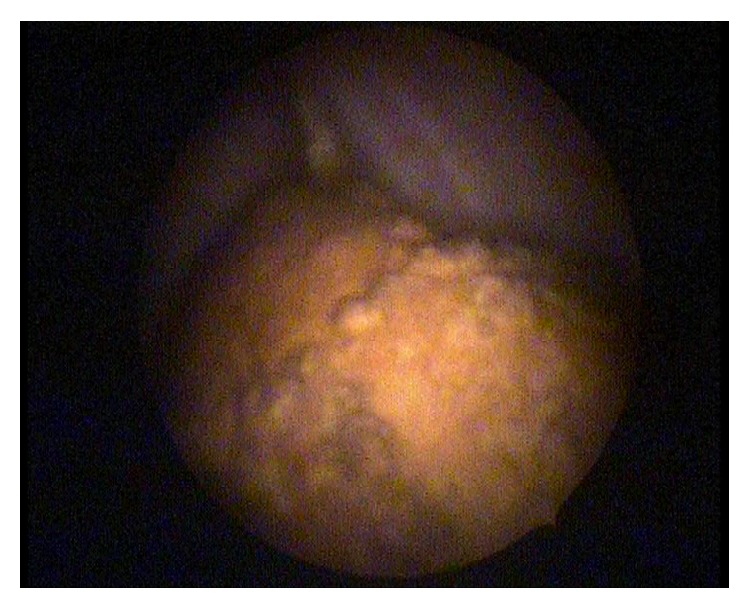
Cystoscopic view of IUD and bladder stone.

**Figure 4:**
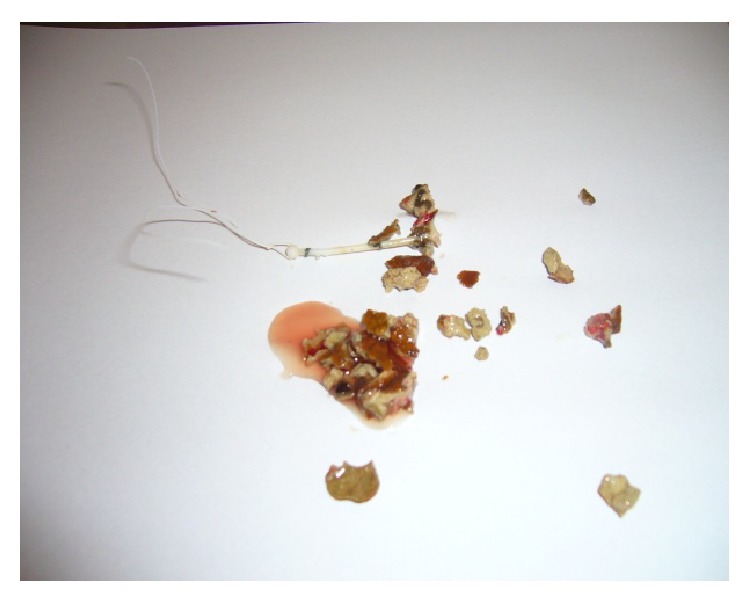
IUD and stone fragments after lithotripsy.

Because of a lost IUD history and abnormal localized hyperechogenic lesion which was suggestive of a foreign body, we had planned a medical consultation to obstetric and gynecology department. Gynecologic examination reported that there were no visible strings of an IUD and cervix was closed. Transvaginal ultrasonography had revealed that more than half of the echogenic foreign body seemed to be in the bladder and a small part of it in the uterovesical space (Figure 1). For confirmation and definitive diagnosis, cystoscopic evaluation was performed under local anesthesia and revealed a partially embedded intravesical IUD on the posterior bladder wall, complicated with a stone formation (Figures 2 and 3). So the patient underwent endoscopic surgery and the stone around the IUD was crushed using holmium laser lithotripsy. After complete disintegration and extraction of fragmented stones, the IUD was removed through the cystoscope using mechanical forceps (Figure 4). The operation time was 50 minutes. The urethral catheter was removed and the patient was discharged on the postoperative day. She was followed up for three months. At the first and third month visit, physical examination and urinalysis were normal.

## 3. Discussion

As being the most widely used reversible contraception method, various complications have accompanied the use of IUD such as uterine perforation and migration to adjacent organs. Albeit they are rare, they can be serious complication. Other than intravesical migration, peritoneum, omentum, rectosigmoid, appendix, small bowel, colon, adnexa, and iliac vein migration were also reported in the literature [7–9]. Migration to these adjacent organs may lead to peritonitis, appendicitis, bowel obstruction and perforation, obstructive nephropathy, infertility due to intraperitoneal adhesions, vesicouterine fistula with menuria, and death due to overwhelming sepsis or pulmonary embolism which have also been reported [5, 10, 11]. Currently, there are about 200 cases of uterine perforation reported (in a literature review in 1999, a total of 165 cases were collected by Kassab and Audra [12]) in the literature. In about 90 of the cases, the IUD migration to bladder was seen with or without stone formation. The true incidence of perforation is most likely higher because of the frequently asymptomatic nature of perforation [13] as a result of misplacement whereas migration due to erosion of the uterus and/or bladder is frequently symptomatic.

The mechanism that causes uterine perforation as a result of migration or misplacement of IUD is not entirely known. But many risk factors associated with uterine perforation as uterine thickness, uterine position, uterine consistency, time of insertion especially in the first 3 months after delivery, congenital uterine anomalies, former pelvic surgeries, and genital infections such as* Actinomyces* [14] have been reported [3]. Additionally, one of the most important factors of perforation is IUD applicator's experience which is probably associated with misplacement; thus, placement of IUD should be performed by experienced ones. With the presence of these risk factors, strong uterine contractions due to delivery or sexual stimulation or spontaneously irregular contraction of bladder, bladder or genital trauma, intestinal motility or peritoneal fluid movement, and accompanying inflammatory effect of IUD may explain the mechanism of gradual migration [15]. In summary, the pathogenesis of uterine perforation by an IUD may occur basically by two mechanisms [5]. First one is perforation at the time of insertion called misplacement and it can be diagnosed by acute pelvic pain, bleeding, or lost thread but most of the perforations at the time of insertion can be overlooked due to asymptomatic nature if not checked by ultrasonography. The second proposed mechanism of uterine perforation may occur gradually and spontaneously after a long time of insertion called IUD migration, in the presence of the risk factors mentioned before with late development of symptoms or being asymptomatic. We suggest that in our case the uterine perforation had occurred at the time of insertion or shortly afterwards, because the patient fell pregnant after three months of insertion of the IUD that proves the loss of contraception or IUD effect in uterus. We also believe that strong uterine contraction due to vaginal delivery should be the major promoting factor of migration in our case. However, a six-year interval of no symptoms also suggested that there should be also other promoting factors such as uterine contractions due to sexual intercourse and inflammatory effect of IUD which can facilitate the migration due to differentiation in tissue consistency. Consequently, we suggested that misplacement of the first IUD was the initiating factor probably associated with perforation; soft consistency of uterus in pregnancy, strong contractions in delivery, and repeatedly sexual intercourse accompanying inflammatory effect of both IUD were the promoting factors probably associated with migration in our case. Thus, patients should be evaluated for the risk factors physically and ultrasonographically before the insertion and examined after the insertion immediately and periodically thereafter for prevention of uterine perforation and other complications [16].

In summary, a patient who has unexpected pregnancy, recurrent urinary infection, LUTS, vesical calculus, and lost tread on self-examination with a history of IUD insertion should be evaluated for IUD perforation and transvesical migration. The initial investigation of choice should include plain abdominal X-ray film since almost all IUD are radio-opaque. An abdominal or transvaginal ultrasound should be performed to detect whether there is transvesical migration or not. By physical examination the IUD perforation or migration can not be excluded, even in the presence of treads on cervical os [17]. If IUD cannot be detected by ultrasound or a second organ injury is suspected, a CT scan will be the proper investigation of choice [18].

## 4. Conclusion

In the current opinion transvesical migration of an IUD with or without stone formation can be treated endoscopically. Also patient expectations necessitate treatment by the least invasive procedure, that is, endoscopically [19]. To the best of our knowledge, it is the third case report of transvesical migrated IUD complicated with stone formation after a transvaginal delivery history, treated with holmium laser lithotripsy. Endoscopic retrieval of the device and stone fragments after application of laser lithotripsy seems to be the least invasive treatment modality for today.

## Abbreviations

CT: Computerized tomography
IUD: Intrauterine device
LUTS: Lower urinary tract symptoms.
